# Cerebral Venous Sinus Thrombosis in Women: Subgroup Analysis of the VENOST Study

**DOI:** 10.1155/2020/8610903

**Published:** 2020-09-01

**Authors:** Derya Uluduz, Sevki Sahin, Taskin Duman, Serefnur Ozturk, Vildan Yayla, Nazire Afsar, Nevzat Uzuner, Ipek Midi, Nilgun Cinar, Mehmet Ali Sungur, Fusun Mayda Domac, Birsen Ince, Baki Goksan, Cemile Handan Misirli, Mustafa Bakar, Hasan Huseyin Kozak, Sena Colakoglu, Ali Yavuz Karahan, Eylem Ozaydin Goksu, Fatih Ozdag, Mehmet Guney Senol, Vedat Ali Yurekli, Ufuk Aluclu, Serkan Demir, Hayriye Kucukoglu, Serdar Oruc, Nilufer Yesilot, Ozge Yimaz Kusbeci, Bijen Nazliel, Firdevs Ezgi Ucan Tokuc, Hesna Bektas, Fatma Nida Tascilar, Emrah Aytac, Mustafa Gokce, Hale Zeynep Batur Caglayan, Ahmet Tufekci, Gulnur Uzuner, Dilek Necioglu Orken, Osman Ozgur Yalin, Uygar Utku, Arda Yilmaz, Hamit Genc, Murat Cabalar, Aysel Milanlioglu, Hakan Ekmekci, Burcu Zeydan, Sevim Baybas, Yuksel Kablan, Basak Karakurum Goksel, Mustafa Acikgoz, Hatice Kurucu, Seden Demirci, Taskin Gunes

**Affiliations:** ^1^School of Medicine, Department of Neurology, Istanbul Cerrahpasa University, Istanbul, Turkey; ^2^School of Medicine, Department of Neurology, Maltepe University, Istanbul, Turkey; ^3^School of Medicine, Department of Neurology, Mustafa Kemal University, Hatay, Turkey; ^4^Selcuk University, School of Medicine, Department of Neurology, Konya, Turkey; ^5^University of Health Science, Hamidiye School of Medicine, Sadi Konuk Research and Training Hospital, Department of Neurology, Istanbul, Turkey; ^6^Acibadem University, School of Medicine, Department of Neurology, Istanbul, Turkey; ^7^Osmangazi University, School of Medicine, Department of Neurology, Eskisehir, Turkey; ^8^Marmara University, School of Medicine, Department of Neurology, Istanbul, Turkey; ^9^Duzce University, School of Medicine, Department of Biostatistics, Duzce, Turkey; ^10^University of Health Science, Hamidiye School of Medicine, Erenkoy Research and Training Hospital for Neurologic and Psychiatric Diseases, Department of Neurology, Istanbul, Turkey; ^11^University of Health Science, Hamidiye School of Medicine, Haydarpasa Training and Research Hospital, Department of Neurology, Istanbul, Turkey; ^12^Uludag University, School of Medicine, Department of Neurology, Bursa, Turkey; ^13^Nemettin Erbakan University, School of Medicine, Department of Neurology, Konya, Turkey; ^14^Usak University, School of Medicine, Department of Physical Medicine and Rehabilitation, Usak, Turkey; ^15^Antalya Research and Training Hospital, Clinic of Neurology, Antalya, Turkey; ^16^University of Health Science, Hamidiye School of Medicine, Sultan Abdulhamid Han Research and Training Hospital, Department of Neurology, Istanbul, Turkey; ^17^Suleyman Demirel University, School of Medicine, Department of Neurology, Isparta, Turkey; ^18^Dicle University, School of Medicine, Department of Neurology, Diyarbakır, Turkey; ^19^Sancaktepe Research and Training Hospital, Clinic of Neurology, Istanbul, Turkey; ^20^Sultan Abdulhamid Research and Training Hospital, Clinic of Neurology, Istanbul, Turkey; ^21^Kocatepe University, School of Medicine, Department of Neurology, Afyon, Turkey; ^22^Istanbul University, School of Medicine, Department of Neurology, Istanbul, Turkey; ^23^Bozyaka Education, Research and Traning Hospital, Clinic of Neurology, Izmir, Turkey; ^24^Gazi University, School of Medicine, Department of Neurology, Ankara, Turkey; ^25^Ataturk Research and Training Hospital, Clinic of Neurology, Ankara, Turkey; ^26^Bulent Ecevit University, School of Medicine, Department of Neurology, Zonguldak, Turkey; ^27^Firat University, School of Medicine, Department of Neurology, Elazig, Turkey; ^28^Sutcu Imam University, School of Medicine, Department of Neurology, Kahramanmaras, Turkey; ^29^Recep Tayyip Erdogan University, School of Medicine, Department of Neurology, Rize, Turkey; ^30^Bilim University, School of Medicine, Department of Neurology, Istanbul, Turkey; ^31^Istanbul Research and Training Hospital, Clinic of Neurology, Istanbul, Turkey; ^32^Mersin University, School of Medicine, Department of Neurology, Mersin, Turkey; ^33^Sadi Konuk Research and Training Hospital, Clinic of Neurology, Istanbul, Turkey; ^34^Yuzuncu Yil University, School of Medicine, Department of Neurology, Van, Turkey; ^35^Mayo Clinic, College of Medicine, Department of Neurology, Rochester, MN., USA; ^36^Bakirkoy Prof. Dr. Mazhar Osman Mental Health and Neurology Training and Research Hospital, Clinic of Neurology, Istanbul, Turkey; ^37^Inonu University, School of Medicine, Department of Neurology, Malatya, Turkey; ^38^Baskent University, School of Medicine, Department of Neurology, Ankara, Turkey; ^39^Akdeniz University, School of Medicine, Department of Neurology, Antalya, Turkey; ^40^Maltepe Government Hospital, Clinic of Neurology, Istanbul, Turkey

## Abstract

**Background:**

Early diagnosis of cerebral venous sinus thrombosis (CVST) associated with reproductive health-related risk factors (RHRF) including pregnancy, puerperium, and oral contraceptive (OC) use can prevent severe neurological sequelae; thus, the symptoms must be documented in detail for each group.

**Methods:**

Out of 1144 patients with CVST, a total of 777 women were enrolled from a multicenter for the study of cerebral venous sinus thrombosis (VENOST). Demographic, biochemical, clinical, and radiological aspects were compared for 324 cases with RHRF and 453 cases without RHRF.

**Results:**

The mean age of the RHRF (-) group (43.2 ± 13 years) was significantly higher than of the RHRF (+) group (34 ± 9 years). A previous history of deep venous thrombosis (3%), isolated cavernous sinus involvement (1%), cranial neuropathy (13%), comorbid malignancy (7%), and its disability scores after 12 months (9%) were significantly higher in the RHRF (-) group. The RHRF (+) group consisted of 44% cases of puerperium, 33% cases of OC users and 23% of pregnant women. The mean age was found to be higher in OC users (38 ± 9 years). A previous history of deep venous thrombosis was slightly higher in the pregnancy subgroup (4%). Epileptic seizures were more common in the puerperium group (44%).

**Conclusion:**

The results of our study indicate that the risk of CSVT increases parallel to age, OC use, and puerperium period. In addition, when considering the frequency of findings and symptoms, epileptic seizures in the puerperium subgroup of the RHRF (+) group and malignancies in the RHRF (-) group may accompany the CSVT. In daily practice, predicting these risks for the CSVT and early recognition of the symptoms will provide significant benefits to patients.

## 1. Introduction

Cerebral venous sinus thrombosis (CVST) is an uncommon form of stroke [1]. Several risk factors for CVST have been recognized including pregnancy, puerperium, oral contraceptive (OC) use, infections, inflammatory diseases, and thrombophilia. CVST is believed to be more common in women than in men [1, 2]. In addition, there is uniform age distribution in men, while 60% of women with CVST are clustered at 20-35 years old [1–4]. In some studies, one of third cases was clustered in periods of pregnancy and puerperium [5].

This study was performed to evaluate details about CVST among women and focused on reproductive health-related risk factors (RHRF) such as pregnancy, puerperium, and OC use.

## 2. Materials and Methods

This study includes 777 female CVST cases of the VENOST cohort. VENOST is a retrospective and multicenter observational study that includes 1144 patients with CVST diagnosed at 35 national neurology centers. In diagnosing CVST, the criteria defined in the VENOST study were used [1].

The patients were divided into two groups according to reproductive health-related risk factors (RHRF) such as oral contraceptive use, puerperium, and pregnancy as the RHRF (+) group and the RHRF (-) group. At the initial admission, both groups were evaluated according to demographics, clinical symptoms, and neurological signs. Radiological workup included brain computed tomography (CT), brain magnetic resonance imaging (MRI), MR venography, and/or digital subtraction angiography. Etiological factors, acute and maintenance treatment, and follow-up results were evaluated for each group. Then, the RHRF (+) group was divided into three subgroups according to risk factors such as oral contraceptive use, puerperium, and pregnancy, and these sub-groups were evaluated using the same risk factors. Putative etiological risk factors included the following: infections (systemic or paracranial infection—otitis media, mastoiditis, or sinusitis), systemic inflammatory diseases, rheumatologic or connective tissue disease, malignancies, and hematologic diseases; and other specified causes were recorded.

The type of onset was considered to be acute if the duration of symptoms was less than 48 hours on admission, subacute if the duration was between 48 hours and 1 month, and chronic if the symptom duration was longer than 1 month. The study was approved by the ethics committee of the coordinating center (Acceptance No. 83045809/604/02-12333).

## 3. Results

In this study, 58% (*n* = 453) of the total 777 female cases were classified as RHRF (-) and 42% (*n* = 324) of them as RHRF (+). The mean ages of the RHRF (+) group and the RHRF (-) group were 34 ± 9 and 43.2 ± 13, respectively, and were significantly different.

Acute onset is more frequent in the RHRF (+) group, whereas a subacute chronic mode of onset is more common in the RHRF (-) group. The most common symptoms were headache, visual field defects, and cranial neuropathies in the RHRF (-) group and headache and epileptic seizure in the RHRF (+) group. The comparison of these two groups according to clinical symptoms and signs: epileptic seizures (34%), nausea and vomiting (33%), and focal neurologic deficit (25%), was more common in the RHRF (+) group and visual field defect (29%) and cranial nerve palsies (13%) were more common in the RHRF (-) group.

In the total female group investigations, CVST was diagnosed with cranial MRI and MRV in 682 patients, with cranial MRI in 46 patients, with only cranial MRV in 31 patients, and with cranial CT and MRV in 14 patients. Parenchymal lesions were detected in 333 (42.8%) female patients including 160 (49.3%) in the RHRF (+) group and 173 (38.1%) in the RHRF (-) group (*p* = 0.003). Parenchimal lesion involvement, especially hemorrhagic transformation (*n* = 80, 25%), was more common in the RHRF (+) group. Venous involvement was found in 1 sinus in 373 (48%) female patients, in 2 sinuses in 274 (35%) patients, and in more than 2 sinuses in 130 (17%) patients. In the comparison of these two groups, there was no difference in intravenous involvement. Transverse sinus involvement was the most common site thrombosis within the total female group (*n* = 572, 73%), within the RHRF (+) group (*n* = 243, 75%), and within the RHRF (-) group (*n* = 329, 73%). The sigmoid sinus and sagittal sinus involvements were followed by transverse sinus in two groups.

Demographic aspects and comparative data of cases with RHRF (+) and RHRF (-) are displayed in Table 1.

A positive previous history of venous thromboembolism and malignancy was detected in 6% and 7% in the RHRF (-) group. Hematological parameters were completed in 206 (26.5%) patients, and no differences were detected between the two groups. When the RHRF (+) group was investigated, it was found to be the largest group in the puerperium period (43.8%) but the smallest group in the pregnancy period (22.8%). Rankin scores, which suggested neurological disability after 12 months, were found significantly high in the RHRF (-) group. Etiological factors and outcome according to groups are presented in Table 2.

The mean age of OC users was higher than other groups. The mode of acute clinical onset was high in all subgroups of RHRF (+) cases. In addition, chronic onset and intracerebral hemorrhage ratio were found more frequently in the OC user group than in the other subgroups. Epileptic seizures were found to be significantly higher in the puerperium group. Demographic and clinical characteristics of subgroup analyses are shown in Table 3.

A history of deep venous thrombosis ratio was found high in the pregnancy group. Hematologic and genetic tests and Ranking scales were similar among the groups. A comparison of etiological factors and outcomes of subgroups is seen in Table 4.

## 4. Discussion

Pregnancy, puerperium, and hormone replacement treatment increase the tendency to cerebral venous sinus thrombosis (CVST) in women. CVST is much more frequently seen in women than in men -a ratio of 3/1 [6]. In the study of Coutinho et al., female ratio was found to be 75% and female gender-specific risk factors at 65% [4]. In the International Study on Cerebral Venous and Dural Sinus Thrombosis (ISCVST), the female ratio was found to be 75% of patients. Gender-specific risk factors such as OCs, pregnancy, puerperium, and hormone replacement therapy were responsible [7]. The results of meta-analyses showed that gender-specific risk factors were only not effective in children and the elderly female groups and that the use of OCs increased venous thrombosis development in reproductive age females [8]. In our study, the female ratio was found to be 68% and gender-specific risks which were grouped as RHRF (+) by us were found in 41% of women. Our findings are similar to the results of previous studies.

In our study, the mean age of women with reproductive health-related risk factors (RHRF) was lower than that of the RHRF (-) group. In subgroup analyses of RHRF (+) cases, the mean age of OC users was higher than that of the other groups. This difference may be related to planning of the age of pregnancy [3].

Previous venous thrombosis history, thrombophilia, certain medical comorbidities, obesity, smoking, and postpartum hemorrhage increase the risk of CVST [9, 10]. In our study, the highest part of the RHRF (+) group consisted of cases of puerperium. Puerperium often occurs in the sixth to eighth week after delivery. In different population-based case-control studies on venous thrombosis, it was explained that risk increased 5-fold in the pregnancy period and a 60-fold in the puerperium [11]. Also, it has been reported that it occurs more commonly after a cesarean birth than a vaginal birth [12]. Infection, high maternal age and excessive vomiting during pregnancy increase the development of CVST [13]. All of the hormone levels, cardiovascular system, and pregnancy-related hematologic changes return to the baseline state within the slow process of puerperium. Human chorionic gonadotropin (hCG) and sex steroids are at low levels for the first 2-3 weeks. These changes may cause the tendency to thrombosis [14–16].

In our study, headache was the most frequent symptom for all subgroups. However, epileptic seizures were higher in the puerperium group. In the study of Kashkoush et al., the highest frequencies of symptom were found to be headache (74%), seizure (50%), and an altered consciousness (45%) in puerperium [17].

The inherited mutations in anticoagulant or thrombolytic factors genes (the Factor V Leiden, the prothrombin Factor II) and mutations in genes coding for proteins C and S may increase the risk of developing venous thrombosis [18]. We did not find any relationship between inherited risk factors and CVST.

Venous thrombosis risk increases with OC use. Combined OCs containing estrogen and progesterone have higher risk [19]. When the patient has a history of previous CVST, the recurrence risk is increased by OC use [20, 21]. We did not determine the content of the OCs. In the RHRF (-) group, a previous history of CVST was high. In the subgroup analysis, a previous history of CVST was high in the “pregnancy group.” Very little is known about the relapse rate during pregnancy and puerperium in women with a history of CVST [22]. The results of our study suggest that physicians must keep in mind the possible recurrence of CVST in pregnancy.

In our study, malignancy was more frequent in the RHRF (-) group. It has been reported that cancer patients have an increased risk of tendency of venous thrombosis [23].

In the study by Lee et al., it has been reported that the transverse sinus is involved in the majority of cases (75.6%). Sigmoid sinus and superior sagittal sinus involvement followed it at ratios of 58.5% and 29.3%, respectively [24]. In our study, the “transverse sinus” was affected more than other venous sinuses. On the other hand, isolated cavernous sinus involvement was significantly high in RHRF (-) group. Cavernous sinus involvement is high in the presence of septicemia and malignancy [25]. Therefore, in our study, this result was expected to be more in the RHRF (-) group.

It has been reported that the prognosis in pregnant patients is better than in nonpregnant patients with CVST if they receive timely treatment [26]. In the VENOST main study, the prognosis of CVST was found to be better in women than men [1]. In our subgroup analysis, the prognosis was found to be worse in the RHRF (-) group.

## 5. Conclusions

Our results indicate that when CVST was detected in women with RHFR (-), the existence of malignancy should be investigated. The previous history of CVST may be related to recurrence in pregnancy. Clinical onset may present with chronic headache in CSVT cases related to OC use. Epileptic seizures may be a more frequent symptom in puerperal CSVT cases. Physicians must keep these situations in mind.

## Figures and Tables

**Table 1:** Compared data of demographic and clinical aspects of groups.

Compared data	RHRF (-)		RHRF (+)		*p*
	*n* = 453		*n* = 324		
Age					
Years	43.2 ± 13	%	34 ± 9	%	<0.001
Mode of onset					
Acute	187^a^	42	195^b^	62	
Subacute	150^a^	34	82^b^	26	<0.001
Chronic	110^a^	25	38^b^	12	
Clinical symptoms and signs					
Isolated headache	119	26	66	20	0.057
Headache	387	85	282	87	0.523
Nausea and vomiting	116	26	107	33	0.024
Epileptic seizures	98	22	110	34	<0.001
Visual field defect	131	29	66	20	0.007
Focal neurological deficit	72	16	81	25	0.002
Altered consciousness	78	17	67	21	0.222
Cranial nerve palsies	59	13	24	7	0.012
Radiological work-up					
Cranial MRI	23	5	23	7	0.492
Cranial MRV	19	4	12	4	
Cranial MRI+MRV	398	88	284	88	
Cranial CT+MRV	10	2	4	1	
Number of sinuses involved					
1 sinus	230	51	143	44	0.281
2 sinuses	148	33	126	39	
More than 2 sinuses	75	16	55	17	
Involved sinuses					
Isolated transverse sinuses	122	27	78	24	0.369
Isolated sagittal sinuses	66	15	44	14	0.697
Isolated sigmoid sinuses	18	4	7	2	0.158
Isolated cortical veins	8	2	11	3	0.147
Isolated jugular sinuses	9	2	1	0	0.052
Isolated cavernous sinuses	6	1	0	0	0.044
Transverse sinuses	329	73	243	75	0.459
Sigmoid sinuses	183	40	127	39	0.736
Sagittal sinuses	157	35	134	41	0.057
Internal jugular vein	71	16	47	15	0.655
Cortical veins	13	3	16	5	0.134
Cavernous sinuses	12	3	3	1	0.085
Parenchymal involvement					
No lesion	280^a^	62	164^b^	51	0.003
Infarction	87^a^	19	66^a^	20	
Hemorrhagic infarction	68^a^	15	80^b^	25	
Intracerebral hemorrhage	18^a^	4	14^a^	4	

MRI: magnetic resonance imaging; MRV: magnetic resonance venography; CT: computed tomography.

**Table 2:** Comparison of etiological factors and outcome according to the RHRF (-) group or the RHRF (+) group.

Compared data	RHRF (-)		RHRF (+)		*p*
	*n* = 453%		*n* = 324%		
Infections					
Paracranial (focal) systemic	20	4	13	4	0.963
	7	2	5	2	
History of VTE					
Cerebral	11^a^	2	2^a^	0.6	
Deep venous thrombosis	14^a^	3	3^b^	1	0.024
Other	6^a^	1	2^a^	0.6	
Malignancy	32	7	1	0.3	<0.001
Family history VTE	5	1	1	0.3	0.409
MTHFR mutation					
Heterozygote	19	7	9	4	0.120
Homozygote	24	8	9	4	
Hyperhomocysteinemia	12	3	9	3	0.952
Prothrombin mutation	5	2	7	3	0.249
Protein C/S deficiency	25	7	13	5	0.302
Factor V Leiden mutation	11	4	11	5	0.405
Thrombocytosis	2	0.5	2	1	0.753
Polycythemia vera	3	1	0	0	0.267
Anticardiolipin Ab	2	0.5	1	0.4	0.752
PAI mutation	4	1	2	1	0.681
Antithrombin III deficiency	3	1	1	0.4	0.642
Hyperfibrinogenemia	0	0	2	1	0.178
Antiphospholipid Ab	7	2	4	2	0.767
Activated protein C	5	1	4	2	0.892
Resistance	13	4	4	2	0.114
High ANA titers					
First month Rankin					
0-1	304	80	236	81	0.276
2	40	11	37	13	
>3	38	10	20	7	
Third month Rankin					
0-1	287	89	227	91	
2	21	7	17	7	0.235
>3	15	5	5	2	
Sixth month Rankin					
0-1	262	90	215	96	
2	16	6	6	3	0.061
>3	13	5	4	2	
12th month Rankin					
0-1	239	91	185	97	
2	11	4	4	2	0.031
>3	13	5	2	1	

ANA: antinuclear antibody; MTHFR: methylenetetrahydrofolate reductase; PAI: plasminogen activator inhibitor; VTE: venous thromboembolism.

**Table 3:** Comparison of demographic and clinical characteristics of subgroups according to reproductive health-related risks.

Compared data of reproductive health-related risk factors	Pregnancy		Puerperium		Oral contraceptive use		*p*
	*n* = 74, 23%		*n* = 142, 44%		*n* = 108, 33%		
Age							
Years	32.2 ± 6^a^		32 ± 7^a^		38 ± 9^b^		<0.001
Mode of onset							
Acute	51^a^	70	85^a^	62	59^a^	56	
Subacute	14^a^	19	42^a^	31	26^b^	25	<0.030
Chronic	8^a,b^	11	10^b^	7	20^a^	19	
Clinical symptoms and signs							
Isolated headache	16	22	24	17	26	24	0.361
Headache	69	93	117	82	96	89	0.062
Nausea and vomiting	22	30	43	30	42	39	0.283
Epileptic seizures	18a	24	63b	44	29a	27	0.002
Visual field defect	17	23	28	20	21	19	0.817
Focal neurological	11	15	41	29	29	27	0.068
Deficit	16	22	34	24	17	16	0.277
Altered consciousness	6	8	8	6	10	9	0.537
Cranial nerve palsies							
Radiological workup							
Cranial MRI	4	6	12	9	7	7	0.975
Cranial MRV	3	4	6	4	3	3	
Cranial MRI+MRV	65	89	122	86	97	90	
Cranial CT+MRV	1	1	2	1	1	1	
Number of sinuses involved							
1 sinus	35	47	68	48	40	37	0.490
2 sinuses	26	35	51	36	49	45	
More than 2 sinuses	13	18	23	16	19	18	
Involved sinuses							
Isolated transverse	21	28	34	24	23	21	0.547
Sinuses	11	15	20	14	13	12	0.838
Isolated sagittal	2	3	3	2	2	1	0.926
Sinuses	1	1	9	6	1	1	0.051
Isolated sigmoid	0	0	1	1	0	0	0.526
Sinuses	0	0	0	0	0	0	—
Isolated cortical veins	57	77	99	70	87	81	0.132
Isolated jugular sinus	30	41	53	37	44	41	0.830
Isolated cavernous	25	34	60	42	49	45	0.284
Sinuses	13	18	17	12	17	16	0.490
Transverse sinuses	2	3	11	8	3	3	0.120
Sigmoid sinuses	0	0	2	1	1	1	0.798
Sagittal sinuses							
Internal jugular vein							
Cortical veins							
Cavernous sinuses							
Parenchymal involvement							
No lesion	48^a^	65	63^b^	44	53^b^	49	0.002
Infarction	12^a^	16	37^a^	26	17^a^	16	
Hemorrhagic	12^a^	16	40^a^	28	28^a^	26	
Infarction	2^a^	3	2^a^	1	10^b^	9	
Intracerebral							
Hemorrhage							

MRI: magnetic resonance imaging; MRV: magnetic resonance venography; CT: computed tomography.

**Table 4:** Etiological factors and outcome of subgroups according to reproductive health-related risks.

Compared data of reproductive health-related risk factors	Pregnancy		Puerperium		Oral contraceptive use		*p*
	*n* = 74%		*n* = 142%		*n* = 108	%	
Infections							
Paracranial (focal)	2	3	4	3	7	7	0.589
Systemic	1	1	3	2	1	1	
History of VTE							
Cerebral	0^a^	0	1a	1	1^a^	1	
Deep venous thrombosis	3^a^	4	0^b^	0	0^b^	0	0.030
Other	0^a^	0	2^a^	1	0^a^	0	
Malignancy	0	0	1	1	0	0	0.526
Family history VTE	1	1	0	0	0	0	0.228
MTHFR mutation							
Heterozygote, homozygote	1	2	5	5	3	5	0.385
Hyperhomocysteinemia	1	2	7	7	1	2	0.204
Prothrombin mutation	0	0	5	4	4	5	0.240
Protein C/S deficiency	0	0	3	3	4	6	0.361
Factor V Leiden mutation	3	5	8	7	2	2	0.335
Thrombocytosis	4	9	3	3	4	6	0.716
Polycythemia vera	1	2	1	1	0	0	0.241
Anticardiolipin Ab	0	0	0	0	0	0	—
PAI mutation	0	0	1	1	0	0	0.517
Antithrombin III deficiency	0	0	1	1	1	2	0.718
Hyperfibrinogenemia	0	0	0	0	1	1	0.568
Antiphospholipid Ab	2	3	0	0	0	0	0.057
Activated protein C	1	2	3	3	0	0	0.362
Resistance	1	2	1	1	2	2	0.821
High ANA titers	0	0	4	4	0	0	0.122
First month Rankin							
0-1	49	78	102	78	85	86	0.177
2	7	11	18	14	12	12	
>3	7	11	11	8	2	2	
Third month Rankin							
0-1	45	92	103	89	79	94	
2	3	6	10	9	4	5	0.828
>3	1	2	3	3	1	1	
Sixth month Rankin							
0-1	44	96	100	94	71	97	
2	1	2	4	4	1	1	0.937
>3	1	2	2	2	1	1	
12th month Rankin							
0-1	34	97	87	95	64	100	
2	1	3	3	3	0	0	0.409
>3	0	0	2	2	0	0	

ANA: antinuclear antibody; MTHFR: methylenetetrahydrofolate reductase; PAI: plasminogen activator inhibitor; VTE: venous thromboembolism.

## Data Availability

All data will be available on request.
